# High affinity monoclonal antibody targeting Siglec-15 for cancer immunotherapy

**Published:** 2021-11-16

**Authors:** Fei He, Na Wang, Jiangwei Li, Luanying He, Zhao Yang, Jiandong Lu, Guoliang Xiong, Changyuan Yu, Shihui Wang

**Affiliations:** ^1^College of Life Science and Technology, Beijing University of Chemical Technology, Beijing 100029, China; ^2^College of Life Science, Key Laboratory of Protection and Utilization of Biological Resources in Tarim Basin of Xinjiang Production and Construction Corps, Tarim University, Alar 843300, Xinjiang, China; ^3^Shenzhen Traditional Chinese Medicine Hospital, Guangzhou University of Chinese Medicine, Shenzhen, 518033, Guangdong, China

**Keywords:** Siglec-15, monoclonal antibody, cancer immunotherapy, hybridoma cell, fluorescent-activated cell sorting

## Abstract

**Background and Aim:**

Recently, Siglec-15 has been proved as a novel immune suppressor and a potential target for normalization cancer immunotherapy, which is non-redundant to the well-known PD-L1/PD-1 pathway. Herein, anti-Siglec-15 mAb, a monoclonal antibody (mAb) with a high affinity against Siglec-15, was prepared.

**Methods:**

The engineered CHO-K1 Siglec-15 cell line was constructed to heterologously expressed Siglec-15 for the affinity test with the mAb. Antigens Siglec-15-mIgG and Siglec-15-his were recombinantly expressed by 293F cells and purified by high-performance liquid chromatography (HPLC). Hybridoma cell line against Siglec-15 was prepared and validated by enzyme-linked immunoabsorbant assay (ELISA) and fluorescent-activated cell sorting (FACS). Finally, the anti-Siglec-15 mAb was produced, purified, and confirmed by SDS-PAGE, ELISA, and FACS.

**Results:**

The EC_50_ of the anti-Siglec-15 mAb with Siglec-15 is 76.65 ng/mL, lower than that of the positive control 5G12 (90.7 ng/mL), indicating a high affinity of the anti-Siglec-15 mAb. *In vitro* and *in vivo* studies verified that the anti-Siglec-15 mAb blocks the Siglec-15-mediated suppression of T cell and moderately prevents the tumor growth.

**Conclusions:**

The anti-Siglec-15 mAb can be considered as an effective immunotherapy for tumor suppression.

**Relevance for Patients:**

The anti-Siglec-15 mAb prepared in this study is useful as an immune checkpoint inhibitor against Siglec-15 for normalization cancer immunotherapy. This immunotherapy provides an alternative treatment for cancer patients who are refractory to the well-known PD-L1/PD-1-targeting therapies.

## 1. Introduction

The sialic-acid-binding immunoglobulin (Ig)-like lectins (Siglecs) are a family of immune-regulatory receptors that are predominantly expressed on the cells of the immune system [1,2]. Siglecs contain an amino-terminal V-set Ig domain that binds sialic acid and variable numbers of C2-set Ig domains, mediating a wide array of recognition and signaling events [3-5]. Up until now, 14 different human Siglecs have been identified and are classified into two subgroups based on sequence homologies: one group containing Siglec-1, Siglec-2, Siglec-4, and Siglec-15, and the other group of all CD33-related Siglecs [6]. Among them, Siglec-15 shows low sequence identity with other Siglecs but is well conserved in mammals throughout vertebrate evolution. Siglec-15 is found to be located on macrophages and/or dendritic cells of human spleen and lymph nodes and composed of two Ig-like domains, a transmembrane domain containing a lysine residue, and a short cytoplasmic tail [7]. The Ig like domains of Singlec-15 are believed to bind preferentially to sialyl-Tn (sTn) (Neu5Acα2-6GalNAcα1-) structure [7,8]. The “immune receptor tyrosine-based inhibition motifs” (ITIM) contained in transmembrane domain could mediate an ITAM-dependent activation through the interactions with the activating adaptor proteins DNAX activation protein 10/12 (DAP10/12) *via* its lysine residue. The evidence implies that Siglec-15 functions as an activating signaling molecule in the immune system of vertebrates [9-11].

The crucial function of Siglec-15 in osteoclast differentiation and bone remodeling has been extensively studied and Siglec-15 was found being highly up-regulated during osteoclast differentiation [9]. Therefore, Siglec-15 monoclonal antibodies (mAbs) have been proposed as a novel type of bone loss therapeutics [12,13]. Recently, several groups have discovered unexpected roles of Siglec-15 in the cancer microenvironment [14]. In general, Siglec-15 is only expressed on some myeloid cells. Now its expression was found to be greatly increased on human cancer cells and/or cancer-associated stromal cells, including tumor-associated macrophages (TAMs) induced by Macrophage colony-stimulating factor (a cytokine inducing alternative activation/polarization of macrophages) [15]. The Siglec-15 preferentially recognizes the sTn antigen, a tumor-associated glycan structure, and the coculture of Siglec-15^+^ myeloid cell line and sTn^+^ cancer cell line enhances the myeloid cell production of transforming growth factor-b (TGF-b) (a pleiotropic cytokine that promotes epithelial-mesenchymal transition and metastasis of cancer cells), which is dependent on DAP12 and SYK [16]. Siglec-15 could also suppress T cell proliferation and activation *in vitro*, and the same effect was found in Siglec-15 deficient mice [17]. These results suggested that Siglec-15 on macrophages may contribute to tumor progression by the TGF-b-mediated modulation of intratumoral microenvironments [18,19].

In a mouse melanoma model, deficiency of Siglec-15 promotes T cell responses, consequently enhancing tumor inhibition and prolonging survival in mice. Siglec-15 targeted by mAbs in wild-type mice reverses the T cell suppression and attenuates cancer growth. In this disease model, Siglec-15 functions as a “ligand” for an unknown inhibitory receptor on cytotoxic T cells, where it acts in a similar manner as PD-L1 (B7-H1, CD274) on tumor cells or tumor stroma involving immune checkpoint molecule PD-1 on T cells [14,20]. A study showed that in mice with whole-body or lineage-specific gene ablation and specific mAbs, Siglec-15 was discovered as an immune suppressive molecule largely operating in the tumor microenvironment (TME) and is non-redundant to the well-known PD-L1/PD-1 pathway. The found effect was partially due to Sigles-15 induction by macrophage colony-stimulating factor and downregulation by IFN-γ. Siglec-15 was also proven to suppress antigen-specific T cell responses *in vitro* and *in vivo*. Genetic ablation or antibody blockade of Siglec-15 amplifies anti-tumor immunity in the TME and inhibits tumor growth in some mouse models. These results collectively demonstrate that Siglec-15 is a potential candidate for normalization cancer immunotherapy [15]. In addition, PD-1 blockade therapy is only appropriate for a small percentage of patients [21]. The expression of siglec-15 (downregulated by IFN-γ) is negatively correlated with the expression of PD-L1 (upregulated by IFN-γ), suggesting that siglec-15 targeted therapy may be a complementary method for cancer patients who are refractory to PD-1/PD-L1-targeting therapies [15]. Currently, a first-in-human phase I clinical trial is ongoing to evaluate the influence of a humanized mAb (NC318) on Siglec-15 in solid tumors (NCT03665285) [22].

In this paper, a mAb with high affinity for Siglec-15, named as anti-Siglec-15 mAb, was screened and produced to facilitate the studies on Siglec-15 targeted therapy and provide a new choice and method for cancer therapy.

## 2. Materials and Methods

### 2.1. Materials

The 293T cells, CHO-K1 cells, lentiviral vectors including pspax2, pMD2G, and pLVX-IRES-Siglec-15-Puro, and Sp2/0 myeloma cells were purchased from Kyinno Biotechnology (Beijing) Co., Ltd, Beijing, China. Dulbecco’s modified Eagle’s medium (DMEM) and F12k medium were bought from Hyclone biotechnology co., LTD, Beijing, China. Fetal bovine serum and Trypsin-EDTA were got from Gibco Laboratories, Beijing, China. Polyetherimide (PEI), puromycin, Freund’s complete adjuvant and water-soluble adjuvant were bought from Solarbio life science co., LTD, Beijing, China. Antibodies PE Goat anti-human IgG Fc was purchased from ThermoFisher scientific co., LTD, Beijing, China. The anti-Siglec-15 antibody 5G12, also known as NC318, was produced by Kyinno Biotechnology (Beijing) Co., Ltd with the patent NO. CN110035769A (PCT, WO2018057735). Hitrap ProteinG HP (1 mL) and HisTrap FF (5 mL) columns were obtained from GE healthcare (Beijing, China). Balb/c mice and NOD-SCID mice (female, 6 – 8-weeks old) were bought from Beijing Biocytogen Co., Ltd (Beijing, China). Enzyme-linked immune sorbent assay (ELISA) kits were purchased from Biolegend Co., Ltd (Beijing, China). The plasmid pMaxGFP™ was bought from BioVector NTCC Inc. (Beijing, China). The Siglec-15-mIgG and Siglec-15-his genes were synthesized by Sangon Biotech Co., Ltd (Shanghai, China). Restriction enzymes *Kpn*I and *Nhe*I were purchased from New England Biolabs (Beijing) Ltd. Human peripheral blood mononuclear cells (PBMCs) were purchased from Fanghui Biotechnology Co., Ltd (Beijing, China). Anti-CD3 mAb (OKT3) was bought from Beifang Tongzheng Biotechnology Co., Ltd (Beijing, China). All other chemicals were of the highest grade commercially available and supplied either by Merch (Beijing, China) or Sigma (Beijing, China).

### 2.2. Construction of Siglec-15-secreting CHO-K1 cells

#### 2.2.1. Lentivirus preparation

**Figure S1:**
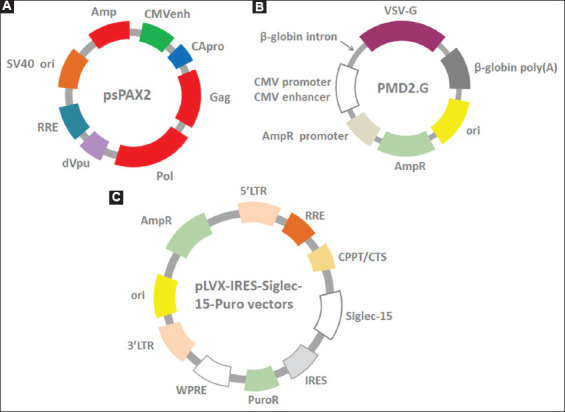
Vectors used for the construction of Siglec-15-secreting CHO-K1 cells. (A) The map of vector psPAX2. (B) The map of vector PMD2.G. (C) The map of vector pLVX-IRES-Siglec-15-Puro.

The 293T cells were cultured in DMEM with 10% fetal bovine serum at 37°C in a humidified 5% CO_2_ atmosphere. Cells were passaged as needed using 0.25% Trypsin-EDTA into fresh medium. An aliquot of 388 μL DMEM was add with 4.5 μL lentiviral vector pspax2 (1 μg/μL), 1.5 μL lentiviral vector pMD2G (1 μg/μL), and 6 μL lentiviral vector pLVX-IRES-Siglec-15-Puro (1 μg/μL) in polypropylene microfuge tube (the maps of these three vectors were seen in Figure S1). The mixture was swirled and incubated for 5 min at room temperature. The mixture was added with 376 μL DMEM and 24 μL PEI (1 μg/μL) and incubated for 10 min. The mixture was gently added into 293T cells dropwise and swirled to disperse evenly. The cells were then incubated at 37°C in a humidified 5% CO_2_ atmosphere. The media were replaced by fresh DMEM 8 h later. After 56-h incubation, the media were harvested from cells, filtered with 0.45 μm membrane, and concentrated with virus concentrate at 4°C overnight. The viruses were subsequently collected by centrifuging the sample at 4°C and 5000 rpm for 45 min. Viruses were stored in DMEM at -80°C before use.

#### 2.2.2. Lentivirus transfection

**Figure S2:**
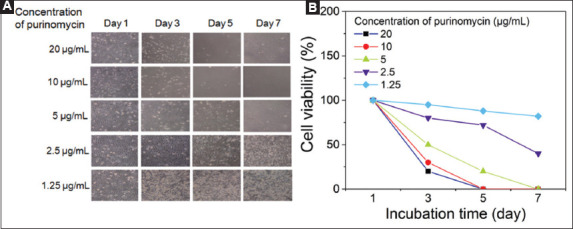
Determination of appropriate puromycin concentration. (A) The growth state of CHO-K1 cells in F12k media under different puromycin concentrations and incubation times. (B) The viability of CHO-K1 cells under different puromycin concentrations and incubation times. The appropriate puromycin concentration was defined as the lowest concentration that kills 100% of the cells in 7 days.

The CHO-K1 cells were cultured in F12k medium at 37°C in a humidified 5% CO_2_ atmosphere. Cells were passaged as needed using 0.25% Trypsin-EDTA into fresh medium. Cells were then adjusted to 2 × 10^5^ cells/mL, plated in the tissue culture plates, and incubated at 37°C in a humidified 5% CO_2_ atmosphere overnight. The cells were added with unfrozen viruses dropwise (100 μL for each well), centrifuged at 1500 rpm for 10 min, and incubated at 37°C in a humidified 5% CO_2_ atmosphere overnight. After 24-h transfection, the medium was replaced with fresh F12k medium containing puromycin at the appropriate concentration. The appropriate puromycin concentration was determined early by incubating CHO-K1 cells under different puromycin concentrations (1.25 – 20 μg/ml) and incubation times (1 – 7 days). The cell viability was monitored at 1^st^, 3^rd^, 5^th^, and 7^th^ day, and 5 mg/ml puromycin was selected as the appropriate concentration as it is the lowest concentration that kills 100% of the cells in 7 days (Figure S2 for detailed information). Every few days, the puromycin-containing media was replaced with fresh ones as needed until the cells were ~100% confluent.

#### 2.2.3. Selection of Siglec-15-secreting CHO-K1 cells by flow cytometry assay

Cells were stained by Trypan blue and counted. An aliquot of 1×10^5^ cells were washed by fluorescent-activated cell sorting (FACS) staining buffer twice. Then cells were added with antibody 5G12, incubated at 4°C for 1 h, and washed by FACS thrice. After that, cells were added with antibody PE Goat anti-human IgG Fc as the secondary antibody (2^nd^ Ab), incubated at 4°C for 1 h, and washed by FACS thrice. The cells were subjected to flow cytometry with Attune NxT (Thermo Fisher Scientific, USA) and analyzed using FlowJo 10. The positive cells expressing Siglec-15 were screened accordingly and expanded in F12k medium containing 5 μg/mL puromycin at 37°C in a humidified 5% CO_2_ atmosphere overnight. A stable CHO-K1 cell line producing Siglec-15 was further selected by the limiting dilution and named as CHO-K1 Siglec-15 cell line. Briefly, the positive cells were plated at 10 cells/mL in F12k media containing 5 μg/mL puromycin in a 96-well plate (100 μL for each well, i.e., 1 cell/well) and incubated at 37°C in a humidified 5% CO_2_ atmosphere. After 5-d incubation, the cells were transferred to a 24-well plate and incubated until the cells were ~100% confluent. The cells were assayed by flow cytometry with Attune NxT following the aforementioned method.

### 2.3. Preparation of Siglec-15-mIgG and Siglec-15-his antigens

**Figure S3:**
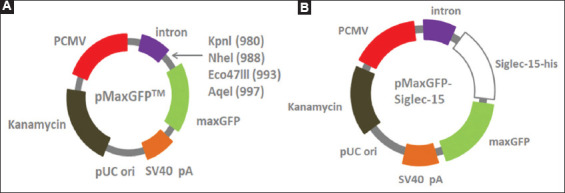
Plasmid construction strategy for heterologous expression of Siglec-15. (A) The map of vector pMaxGFP™; (B) The map of pMaxGFP™ with the insertion of Siglec-15-mIgG or Siglec-15-his (pMax1-Siglec-15-mIgG or pMax1-Siglec-15-his) cut by restriction enzymes *Eco*47III and *Nhe*1.

**Figure S4:**
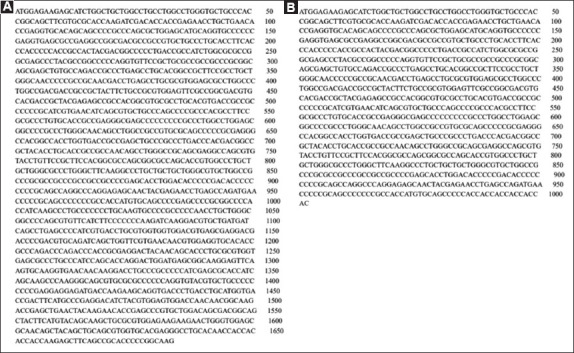
The coding sequence of Siglec-15-mIgG (A) and Siglec-15-his genes (B)

The plasmid pMaxGFP™ and exogenous genes Siglec-15-mIgG or Siglec-15-his were cut by restriction enzymes *Eco*47III and *Nhe*1 and linked by T4 DNA Ligase to obtain recombinant plasmids pMax1-Siglec-15-mIgG and pMax1-Siglec-15-his. The plasmid construction strategy and the sequences of Siglec-15-mIgG and Siglec-15-his were shown in Figures S3 and S4. Subsequently the plasmids pMax1-Siglec-15-mIgG and pMax1-Siglec-15-his were amplified in *Escherichia coli* and transient transfected into 293F cells. Then the antigens Siglec-15-mIgG and Siglec-15-his were expressed recombinantly in 293F cells. The Siglec-15-mIgG was purified by Hitrap ProteinG HP column using the AKTA explorer (GE healthcare, Beijing, China). The equilibrium buffer was 150 mM phosphate-buffered saline (PBS) (pH 7.2) and the elution buffer was 100 mM glycine-HCl buffer (pH 2.7). The Siglec-15-his was purified by HisTrap FF column using the AKTA explorer. The equilibrium buffer contained 20 mM sodium phosphate, 0.5 mM NaCl, and 30 mM imidazole (pH 7.4). The elution buffer contained 20 mM sodium phosphate, 0.5 mM NaCl, and 200 mM imidazole (pH 7.4). The purified Siglec-15-mIgG and Siglec-15-his solutions were neutralized by 1 M Tris-HCl (pH 9). The Siglec-15-mIgG and Siglec-15-his antigens were verified by SDS-PAGE and ELISA using commercial ELISA kits.

### 2.4. Preparation of anti-Siglec-15 mAb

#### 2.4.1. Mice administration and ELISA assay of anti-serum

Immunized by Freund’s complete adjuvant: Siglec-15-mIgG was used as an immune antigen. Five Balb/c mice were administered primarily with Siglec-15-mIgG (2 mg/mL) added with an equivalent volume of Freund’s complete adjuvant at multiple sites subcutaneously. Booster injections of Siglec-15-mIgG added with an equivalent volume of Freund’s incomplete adjuvant were given at 2-week intervals. After 5 times administration, blood from each mouse was harvested sequentially from the tail vein [23,24].

Immunized by water-soluble adjuvant: The method was the same with the “Freund’s complete adjuvant” except that the Freund’s complete adjuvant was replaced by water-soluble adjuvant.

The individual anti-sera were assayed by ELISA. Briefly, 96-well ELISA plate was coated with BSA (50 ng/well), and incubated overnight at 4°C. Plate was washed three times with PBS, blocked with PBSM (5% skim milk powder in PBS, 200 μL/well), and incubated for 2 h at 37°C. Later, the plate was washed three times with PBS and PBST (0.5% Tween 20 in PBS). Mice serum was added to the plate, and incubated for 2 h at 37°C. After sufficient washing, goat anti-mouse IgG-HRP (1:10000, 100 μL/well) was added and incubated for 1 h at 37°C. After washing, tetramethylbenzidine, a chromogenic reporter, was added at 50 μL/well and incubated in the dark for 15 min at 37°C. Then 2 M H_2_SO_4_ (50 μL/well) was added to stop the reaction mixture and a yellow reaction product was formed after acidification that was measured at 450 nm by microplate reader to quantitatively determine the anti-sera [24].

#### 2.4.2. Cell fusion and screening of anti-Siglec-15 mAb

Hybridoma cell line was developed against Siglec-15 based on a standard modified method [24]. The anti-serum of the immunized mouse with highest anti-Siglec-15 antibody titer was chosen and executed before the cell fusion. The splenocytes from administered mice were harvested and fused with Sp2/0 myeloma cells (grown in three cell culture dishes) at relative ratio at 1:10 in the presence of 50% PEG-2000 (1 mL) added dropwise. Hybridoma cells were thoroughly cultured, HAT selected and subsequently screened for anti-Siglec-15 pAb by ELISA. For the ELISA, the Siglec-15-his was used as the antigen, the anti-serum was served as the primary antibody, and the HRP Goat anti-mouse IgG Fc was served as the secondary antibody.

Sub-cloning was carried out by limiting dilution method for subsequent screening of positive hybridoma clone and expanded. Positive hybridoma cells producing anti-Siglec-15 mAb were screened by FACS similar to the aforementioned method. Briefly, the CHO-K1 Siglec-15 cells were passaged as needed, adjusted to 4 × 10^6^ cells/ml in FACS buffer, and injected into the flow cytometry micro well plates (50 μL/well). Cells were added with the anti-serum of the positive hybridoma clone, incubated for 1 h at 4°C, and washed 3 times with FACS buffer. PE Goat anti-mouse IgG Fc was added to the plate (5 μL/well), incubated for 1 h at 4°C, and washed 3 times with FACS buffer. The sample was then subjected to the flow cytometry assay to select positive hybridoma cells producing anti-Siglec-15 mAb.

#### 2.4.3. Production, purification, and analysis of anti-Siglec-15 mAb

Positive hybridoma cells producing anti-Siglec-15 mAb were expanded to obtain 100 mL broth. The broth was centrifuged and filtered by 0.45 μm membrane to obtain the supernatant. The anti-Siglec-15 mAb was purified from the supernatant by Hitrap ProteinG HP following the aforementioned instructions. The concentration of the mAb was determined by NanoDrop (Thermo Fisher Scientific, Beijing, China). The mAb was validated by SDS-PAGE, ELISA, and FACS following the methods described above [23].

### 2.5. In vitro and in vivo efficiency of anti-Siglec-15 mAb

For the *in vitro* efficiency of anti-siglec-15 mAb, T cell response assays were conducted by stimulating human PBMCs with 0.1 μg/ml anti-CD3 in the presence of 5 μg/ml Siglec-15 with or without anti-Siglec-15 mAb at 12 μg/ml. Negative control contained mIgG instead of Siglec-15. Positive control contained 5G12 instead of anti-Siglec-15 mAb. Blank control contained medium only. The proliferation of CD4+ T cell and CD8+ T cell was determined by CFSE dilution.

For the *in vivo* efficiency of anti-siglec-15 mAb, H157 tumor cells and PBMCs were mixed at the ratio of 4:1 and subcutaneously injected into NOD-SCID mice at 5×10^6^ per mouse (*n*=8 mice per group). At day 1, 4, 8, and 12, mice were treated intraperitoneally with anti-Siglec-15 mAb (100 or 200 μg). Tumor growth in the mice was monitored by measuring the mean tumor diameter with a caliper at indicated days and presented as the mean tumor diameter (length + width)/2 in mm.

### 2.6 Statistical analyses

The results were expressed as the mean ± SEM. SPSS 19 was used to perform the statistical analysis and Origin 8 software was used for graphical plotting of data. Comparisons of all data were made using Student’s t-test and one-way analysis of variance (ANOVA). *P*<0.05 were considered statistically significant. *P<0.05, ** *P*<0.01, *** *P*<0.001, and ****P<0.0001.

## 3. Results and Discussion

### 3.1. Construction of CHO-K1 Siglec-15 cell line

**Figure 1:**
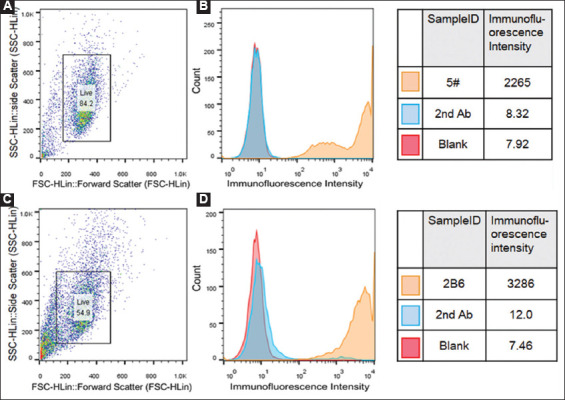
Construction of CHO-K1 Siglec-15 cell line. (A and B) Primary screening of CHO-K1 Siglec-15 cell lines by flow cytometry. (C and D) Secondary screening of CHO-K1 Siglec-15 cell lines by flow cytometry. The primary antibody was 5G12 and the secondary antibody (2^nd^ Ab) was PE Goat anti-human IgG Fc. Cells were stained by PE. The 2^nd^ Ab was used as the negative control. In Figure 1B and 1D, the cells expressing Siglec-15 were counted (Y axis), and the yellow fluorescence intensity (X axis) was positively related to the Siglec-15 expression.

The engineered CHO-K1 cell line producing Siglec-15 was constructed by lentivirus infection and selected by flow cytometry assay. As illustrated in Figure 1A and B, cell line 5# showed the highest fluorescence intensity of all the cells, which is ca. 300 folds of that from the blank control and was therefore selected as the positive cell line producing Siglec-15. Then cell line 5# was further expanded and diluted to 1 cell/well in a 96-well plate to screen a stable and Siglec-15 high-yield CHO-K1 cell line. As can be seen in Figure 1C and D, the cell line 2B6 shows the highest fluorescence intensity, therefore it was picked as the positive Siglec-15-producing cell line for future use and named as CHO-K1 Siglec-15 cell line.

### 3.2. Preparation of Siglec-15-mIgG and Siglec-15-his antigens

**Figure 2:**
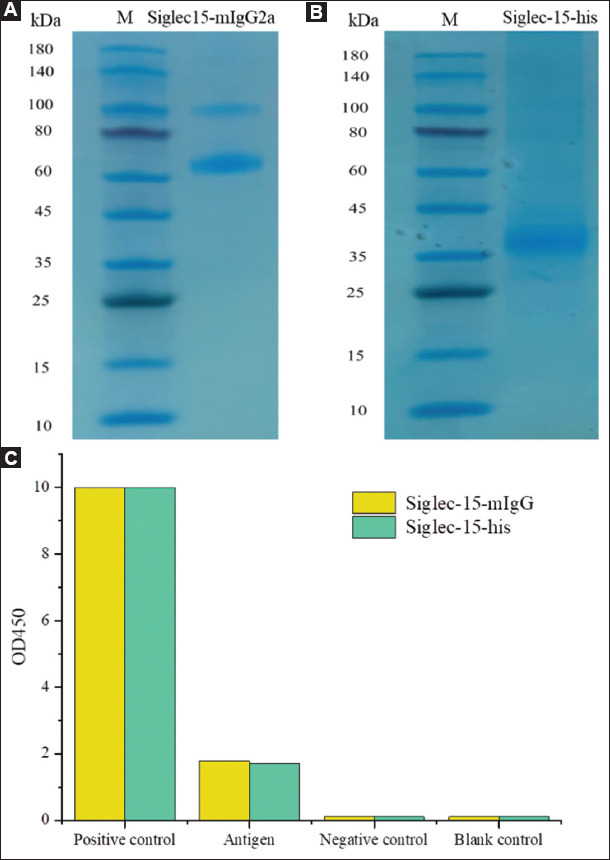
The characterization of Siglec-15-mIgG and Siglec-15-his antigens. (A and B) The SDS-PAGE assays of Siglec-15-mIgG and Siglec-15-his. (C) The ELISA results of Siglec-15-mIgG and Siglec-15-his. The positive control was 5G12 and the negative control was mIgG.

Antigen Siglec-15-mIgG, containing mFc tag, was planned to be used for immunization. The addition of mFc tag increases the mass and volume of the antigen, therefore elevates the half-life period of antigen *in vivo*. The longer half-life period is supposed to enhance the performance of antigen and improve the titer of antibody. Besides, the mFc tag facilitates the purification of Siglec-15-mIgG by an affinity chromatography with G-protein columns (Hitrap ProteinG HP column) [25]. Antigen Siglec-15-his, including his tag, was aimed for screening of anti-Siglec-15 mAb. Siglec-15-mIgG and Siglec-15-his were separately and recombinantly expressed in 293F cells and purified by high-performance liquid chromatography (HPLC) for the generation and selection of anti-Siglec-15 mAb. The SDS-PAGE results of Siglec-15-mIgG and Siglec-15-his were shown in Figure 2A and B. The molecular weights of Siglec-15-mIgG and Siglec-15-his are 65 and 38 kDa, respectively, which is consistent with the values reported in the literature [12]. The affinity and specificity of the two antigens were verified by ELISA using anti-human Siglec-15 antibody (namely 5G12) as the primary antibody and HRP Goat anti-human IgG Fc as the secondary antibody. As shown in Figure 2C, the OD450 of Siglec-15-mIgG and Siglec-15-his are 1.78 and 1.72. By comparing the results from positive, negative, and blank control, it can be concluded that the affinity of the two antigens with the anti-human Siglec-15 is high enough for the further preparation and screening of anti-Siglec-15 mAb.

### 3.3. Selection of hybridoma cell line producing anti-Siglec-15 mAb

**Figure 3:**
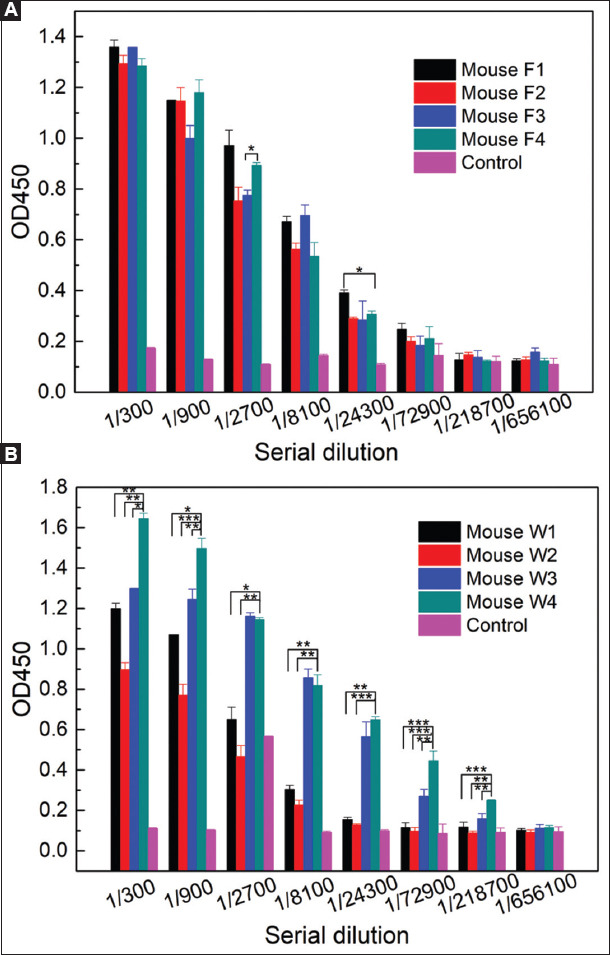
Selection of the immunized mouse producing anti-Siglec-15 mAb. (A) Serum titer of Balb/c mice immunized by Freund’s complete adjuvant. (B) Serum titer of Balb/c mice immunized by water-soluble adjuvant. The number behind F and W denotes the mouse number in the experiments. All the mice were administered 5 times by Siglec-15-mIgG. * denotes *p* compared between different mice (*P<0.05, ** *P*<0.01, *** *P*<0.001, *n*=5).

**Figure 4:**
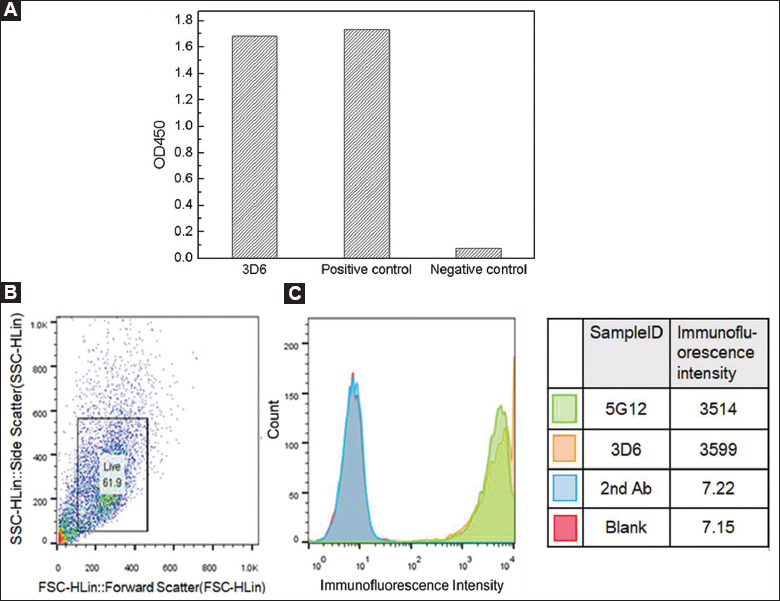
Screening of positive hybridoma cell line secreting anti-Siglec-15 mAb by ELISA (A) and flow cytometry (B and C). The positive control was 5G12 and the negative control was 2^nd^ Ab. For ELISA (A), the primary antibody was anti-serum and the secondary antibody (2^nd^ Ab) was HRP Goat anti-mouse IgG Fc. For flow cytometry (B, C), the primary antibody was anti-serum and the secondary antibody (2^nd^ Ab) was PE Goat anti-mouse IgG Fc. Cells were stained by PE. In Figure 4C, the cells secreting anti-Siglec-15 mAb were counted (Y axis), and the yellow fluorescence intensity (X axis) was positively related to the Siglec-15 secreting.

The mice were immunized by Siglec-15-mIgG through Freund’s complete adjuvant and water-soluble adjuvant, respectively. The titer of the anti-serum from the immunized mice was tested by indirect ELISA (iELISA) and the results were shown in Figure 3A and B. The OD450 values of the blood sample from all the Balb/c mice are significantly higher than that of the control, suggesting that the expression of antibody against Siglec-15 was successfully induced by Siglec-15-mIgG. The mouse W4 immunized by water-soluble adjuvant showed the highest titer of 656100 (Figure 3B) (*P*<0.05). Therefore, the spleen cells of mouse W4 was selected to perform cell fusion experiments with Sp2/0 myeloma cells. The supernatant of growing hybridoma cells was tested by iELISA, and the positive hybridoma clones were picked. Five positive hybridoma clones with the highest OD450 values were selected for subsequent screening of sub-clones. Sub-cloning was carried out by limiting dilution method in five 96-well plates and the hybridoma cell line 3D6, with the highest OD450, was selected for further verification (Figure 4A).

The affinity and specificity of the anti-serum produced by hybridoma cell line 3D6 with the CHO-K1 Siglec-15 cells were tested by flow cytometry and the results were shown in Figure 4B. It can be seen that the fluorescence intensity of the cell line 3D6 is similar to that of the positive control (5G12), and also much higher than that of the secondary antibody and blank control, suggesting that the hybridoma cell line 3D6 was successfully screened to produce anti-Siglec-15 mAb.

### 3.4. Production, purification and evaluation of anti-Siglec-15 mAb

**Figure 5:**
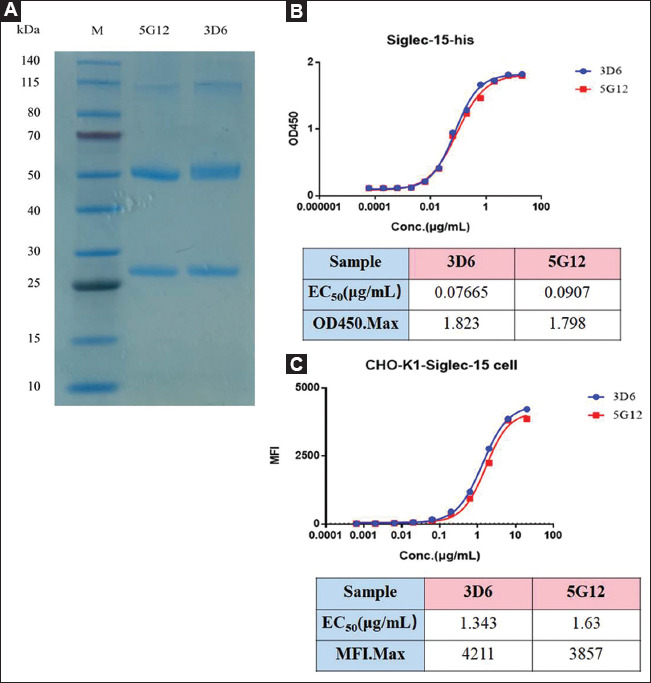
The characterization of anti-Siglec-15 mAb produced by hybridoma cell line 3D6. (A) SDS-PAGE of 5G12 and 3D6. (B) Affinity of anti-Siglec-15 mAb with Siglec-15-his determined by ELISA. (C) Affinity of anti-Siglec-15 mAb with CHO-K1 Siglec-15 determined by ELISA. For ELISA (B and C), the primary antibody was anti-serum and the secondary antibody (2^nd^ Ab) was HRP Goat anti-mouse IgG Fc. 5G12 was used as the positive control.

The hybridoma cell line 3D6 was expanded *in vitro* to obtain 100 mL anti-serum. The anti-Siglec-15 mAb was purified from the anti-serum through HPLC and assayed by SDS-PAGE. The results showed that the heavy chain of the mAb is 50 kDa and the light chain is 27 kDa, which are consistent with the control 5G12 (Figure 5A). It indicated that the anti-Siglec-15 mAb was successfully purified.

Affinity of the anti-Siglec-15 mAb with Siglec-15-his was determined by ELISA using 5G12 as the positive control. As shown in Figure 5B, the EC50 of the anti-Siglec-15 mAb is 76.65 ng/mL, lower than that of the positive control 5G12 (90.7 ng/mL), indicating that the affinity between anti-Siglec-15 mAb and Siglec-15 is higher than the positive control.

Affinity between the anti-Siglec-15 mAb and CHO-K1 Siglec-15 cells was evaluated by flow cytometry assay using 5G12 as the positive control. The results in Figure 5C showed that the EC_50_ of the anti-Siglec-15 mAb is 1.343 μg/mL, which is lower than that of the positive control 5G12 (1.63 μg/mL)) indicating that the affinity between anti-Siglec-15 mAb and CHO-K1 Siglec-15 cells is higher than the positive control.

The above results collaboratively proved that the anti-Siglec-15 mAb produced by the hybridoma cell line 3D6 possesses a high affinity with Siglec-15, indicating that it could be applied on the research and application for normalization cancer immunotherapy.

### 3.5. In vitro and in vivo efficiency of anti-Siglec-15 mAb

**Figure 6:**
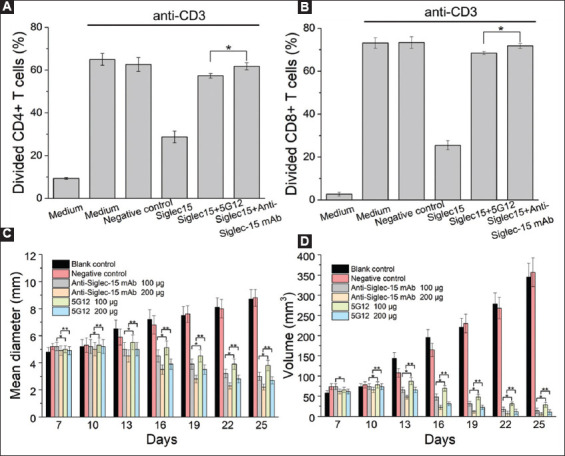
*In vitro* and *in vivo* efficiency of anti-Siglec-15 mAb. (A and B) Human PBMCs were stimulated by 0.1 μg/ml anti-CD3(OKT3) in 96-well plates for 3 days in the presence of 5 μg/ml Siglec-15 with or without anti-Siglec-15 mAb at 12 μg/ml. The proliferation of CD4+ T cell (A) and CD8+ T cell (B) was determined by CFSE dilution. Blank control contained medium only. Negative control contained mIgG instead of Siglec-15. Positive control contained 5G12 instead of anti-Siglec-15 mAb. (C and D) H157 tumor cells and PBMCs were mixed at the ratio of 4:1 and subcutaneously injected into NOD-SCID mice at 5×10^6^ per mouse and subsequently treated with anti-Siglec-15 mAb (100 or 200 μg) at day 1, 4, 8, and 12 (*n*=8 mice per group). Tumor mean diameters (C) and volumes (D) were obtained every 3 days. PBS, mIgG, and 5G12 were used as blank control, negative control, and positive control, respectively. * denotes *p* compared between different groups (*P<0.05, ** *P*<0.01, *** *P*<0.001, *n*=5).

The influence of anti-Siglec-15 mAb on the proliferation of T cells was explored using 5G12 as a positive control. As shown in Figure 6A and B, Siglec 15 inhibits the proliferation of CD4+ and CD8+ T cells. The addition of anti-Siglec-15 mAb blocked the Siglec-15-mediated suppression of T cell, and the efficiency of it is better than that of 5G12 (*P*<0.05).

We then tested the effect of anti-Siglec-15 mAb on an established NOD-SCID mouse model (Figure 6C and D). Seven days after tumor cell inoculation, tumors were well established in syngeneic mice according to the tumor volumes. Treatment by anti-Siglec-15 mAb or 5G12 at 100 and 200 mg induces a moderate suppression on the growth of tumors. The efficacy of anti-Siglec-15 mAb is better than that of 5G12 (100 mg, *P*<0.05; 200 mg, *P*<0.01), which agrees with the *in vitro* results (Figure 6A and B).

## 4. Conclusion

We herein developed an anti-Siglec-15 mAb against Siglec-15 with the EC_50_ value of 76.65 ng/mL. First, the CHO-K1 Siglec-15 cell line was constructed to express abundant Siglec-15. Then antigens Siglec-15-mIgG and Siglec-15-his were expressed recombinantly by 293F cells and purified by HPLC. Hybridoma cell line secreting anti-Siglec-15 mAb was prepared and verified by ELISA and FACS. Finally, the anti-Siglec-15 mAb was produced, purified, and validated by SDS-PAGE, ELISA, and FACS. Both *in vitro* and *in vivo* studies confirmed that the anti-Siglec-15 mAb blocks the Siglec-15-mediated suppression of T cell and moderately inhibits the tumor growth. Thus, we concluded that this anti-Siglec-15 mAb is a very useful tool in evaluating Siglec-15 targeted therapy in the preclinical setting for tumor treatment.

### Ethics Approval and Consent to Participate

Not applicable.

### Human and Animal Rights

The authors declare that the procedures followed were in accordance with the regulations of the responsible Clinical Research Ethics Committee and in accordance with those of the World Medical Association (protocol number: 2018S016).

### Conflict of Interest

The authors declare no conflict of interest.
